# Constipation in Adults and Children

**Published:** 1898-07

**Authors:** 


CONSTIPATION IN ADULTS AND CHILDREN. With special reference to Habitual Constipation and its Most Successful Treatment by the Mechanical Methods. By H. Illoway, M. D., formerly Professor of the Diseases of Children, Cincinnati Medical College, etc. Illustrated. Published by the Macmillan Co., New York. 1897. Pages, 495. Price, cloth, $4.

Another good book. The subject is taken up systematically, beginning with the anatomy and physiology of the intestinal tract, and every detail of interest and value is thoroughly discussed. Over two hundred pages are occupied in discussing treatment, every phase of which receives proper consideration. We are somewhat surprised at the amount of valuable information to be had upon the subject, and congratulate the author upon his valuable contribution to medical literature. J.

